# Possible effects of mixed prevention strategy for COVID-19 epidemic: massive testing, quarantine and social distancing

**DOI:** 10.3934/publichealth.2020040

**Published:** 2020-07-06

**Authors:** Toshikazu Kuniya, Hisashi Inaba

**Affiliations:** 1 Graduate School of System Informatics, Kobe University, 1-1 Rokkodai-cho, Nada-ku, Kobe 657-8501, Japan; 2 Graduate School of Mathematical Sciences, The University of Tokyo, 3-8-1 Komaba, Meguro-ku, Tokyo 153-8914, Japan

**Keywords:** COVID-19, asymptomatic transmission model, control reproduction number, state-reproduction number

## Abstract

**Background:**

The pandemic coronavirus disease 2019 (COVID-19) has spread and caused enormous and serious damages to many countries worldwide. One of the most typical interventions is the social distancing such as lockdown that would contribute to reduce the number of contacts among undiagnosed individuals. However, prolongation of the period of such a restrictive intervention could hugely affect the social and economic systems, and the outbreak will come back if the strong social distancing policy will end earlier due to the economic damage. Therefore, the social distancing policy should be followed by massive testing accompanied with quarantine to eradicate the infection.

**Methods:**

In this paper, we construct a mathematical model and discuss the effect of massive testing with quarantine, which would be less likely to affect the social and economic systems, and its efficacy has been proved in South Korea, Taiwan, Vietnam and Hong Kong.

**Results:**

By numerical calculation, we show that the control reproduction number is monotone decreasing and convex downward with respect to the testing rate, which implies that the improvement of the testing rate would highly contribute to reduce the epidemic size if the original testing rate is small. Moreover, we show that the recurrence of the COVID-19 epidemic in Japan could be possible after the lifting of the state of emergency if there is no massive testing and quarantine.

**Conclusions:**

If we have entered into an explosive phase of the epidemic, the massive testing could be a strong tool to prevent the disease as long as the positively reacted individuals will be effectively quarantined, no matter whether the positive reaction is pseudo or not. Since total population could be seen as a superposition of smaller communities, we could understand how testing and quarantine policy might be powerful to control the disease.

## 1 Introduction

Coronavirus disease 2019 (COVID-19) was first identified in Wuhan, China on December 2019 [1]. The World Health Organization (WHO) characterized COVID-19 as a pandemic on 11 March, 2020 [1], and it has caused enormous and serious damages to the health, medical, social and economic systems in many countries worldwide. As of 1 July, 2020, 10,357,662 people have been reported to be infected by COVID-19, and 508,055 people passed away due to COVID-19 [1].

**Figure 1.**
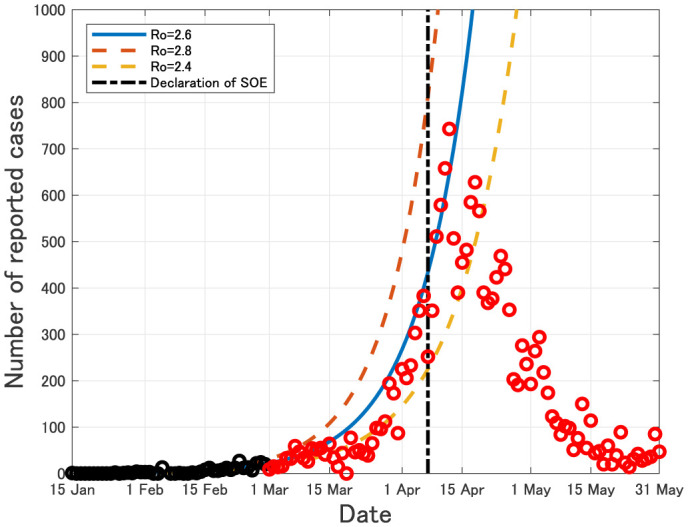
Daily number of newly reported cases in Japan from 15 January to 31 May, 2020. Black circles imply the data from 15 January to 29 February, 2020, which were used for estimating the epidemic curves (see also [2]). Red circles imply the data after 1 March, 2020. *R*_0_ is the basic reproduction number [3].

In Japan, the first case of COVID-19 was identified on 15 January, 2020 [1]. As of 1 July, 2020, the number of total reported cases of COVID-19 in Japan has reached to 18,723 and that of total deaths is 974 [1]. In [2], the author estimated the epidemic parameters for COVID-19 in Japan by using the data from 15 January to 29 February, 2020 [1]. The estimated epidemic curves seem to fit the actual data before 20 April (see Figure 1).

The state of emergency (SOE) in Japan was first declared on 7 April, 2020 for 7 prefectures (Tokyo, Kanagawa, Sitama, Chiba, Osaka, Hyogo and Fukuoka), and it was then expanded to all 47 prefectures on 16 April, 2020. From Figure 1, we see that the daily number of newly reported cases of COVID-19 in Japan has tended to decrease since about 2 weeks passed from the first state of emergency.

**Figure 2.**
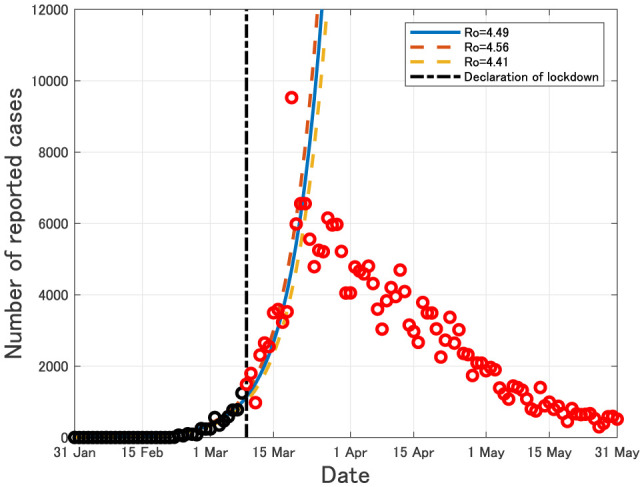
Daily number of newly reported cases in Italy from 31 January to 31 May, 2020. Black circles imply the data from 31 January to 8 March, 2020, which were used for estimating the epidemic curves. Red circles imply the data after lockdown on 9 March, 2020.

In Italy, the rapid exponential growth of the daily number of newly reported cases of COVID-19 was observed in late February, 2020, and the lockdown has started from 9 March, 2020. After about 2 weeks passed from the lockdown, the daily number of newly reported cases of COVID-19 in Italy has tended to decrease as of 31 May, 2020 (see Figure 2).

As is reported in many references (see, e.g., [4]–[8]), not a few individuals infected by COVID-19 are asymptomatic. Therefore, the social distancing such as lockdown would be one of the most effective ways to control COVID-19 because it would contribute to reduce the number of contacts among undiagonosed individuals. However, the prolongation of the period of such a restrictive intervention could hugely affect the social and economic systems, its financial and psychological cost is too high.

**Figure 3.**
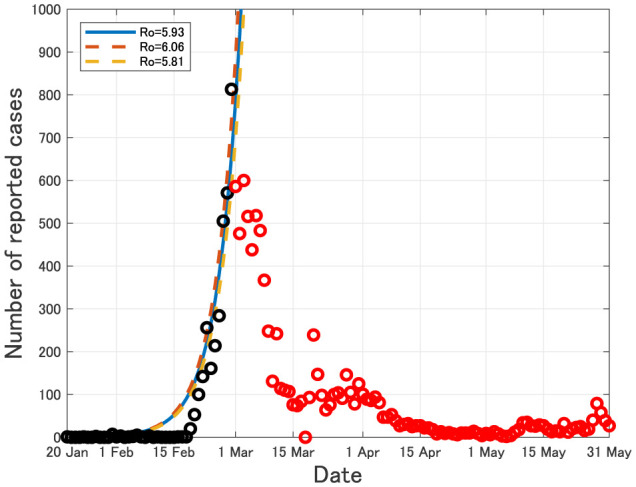
Daily number of newly reported cases in South Korea from 20 January to 31 May, 2020. Black circles imply the data from 20 January to 29 February, 2020, which were used for estimating the epidemic curves. Red circles imply the data after 1 March, 2020.

In South Korea, COVID-19 has been successfully controlled without lockdown as of 31 May, 2020 (see Figure 3).

One of the remarkable differences between South Korea and Japan in the early stage was the proactivity for testing. As of 20 April, 2020, the total number of reported cases for COVID-19 in Japan (10,751) is almost the same as that in South Korea (10,674) [1], however, the total number of COVID-19 tests per 1,000 people in South Korea (10.98) is about 7 times larger than that in Japan (1.58) [9]. Since COVID-19 has high infectivity before symptom onset [10], testing would be one of the most effective ways to reduce the number of contacts among individuals with no symptoms. In particular, we can expect that massive testing would be less likely to affect the social and economic systems because it does not require any strong restrictions on the personal behavior. The efficacy of testing has been proved also in Taiwan, Vietnam and Hong Kong [9].

In this paper, we discuss the possible effects of social distancing and massive testing with quarantine by constructing a mathematical model, which is based on the classical SEIR epidemic model (see, e.g., [11], [12] for previous studies on the control effect for SEIR epidemic models). The organization of this paper is as follows. In section 2, we formulate the basic asymptomatic transmission model to derive the control reproduction number *R_c_* and the state-reproduction number. In section 3, we estimate the baseline parameters and examine the effects of social distancing and massive testing accompanied with quarantine by numerical simulation. In section 4, we briefly review the outcome of the early control strategy for COVID-19 and discuss the feasibility of the massive testing.

## 2 Materials and method

### 2.1 Basic asymptomatic transmission model

**Figure 4.**
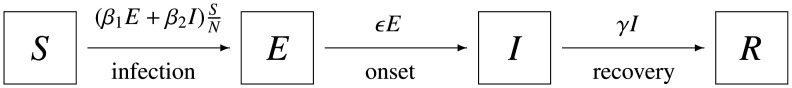
Transfer diagram for model (1).

Our basic model is a well-known SEIR epidemic model [3] with standard incidence, though it is extended to recognize the asymptomatic transmission. In order to focus on the effect of comtrol measures, for simplicity, we neglect the additional death due to the epidemic. If the total size of host population is so large, this assumption would be irrelevant to our conclusions. Let *S* be the susceptibles, *E* the asymptomatic infecteds, *I* the symptomatic infecteds, *R* the recovereds. Then the basic dynamics without intervention is formulated as follows (see also Figure 4):

$$\begin{array}{l} \frac{dS}{dt}=-\left(\beta_{1}E+\beta_{2}I\right)\frac{S}{N}\\ \frac{dE}{dt}=\left(\beta_{1}E+\beta_{2}I\right)\frac{S}{N}-\varepsilon E\\ \frac{dI}{dt}=\varepsilon E-\gamma I\\ \frac{dR}{dt}=\gamma I \end{array}$$ (1)

where *β*_1_ denotes the asymptomatic transmission rate, *β*_2_ the symptomatic transmission rate, *ε* the onset rate, *γ* the recovery rate for infecteds. Under this assumption, the total population size *N*:= *S* + *E* + *I* + *R* is constant. Therefore, we can interpret each population size as the prevalence of each status if we set *N* = 1. Note that if *β*_1_ = 0 and *β*_2_ > 0, then (1) is the usual SEIR model without asymptomatic transmission. The asymptomatic transmission is taken into account only if *β*_1_ > 0.

The linearized system at the disease free steady state for (1) is

$$\frac{dx\left(t\right)}{dt}=\left(B+C\right)x\left(t\right)$$ (2)

where *x*(*t*):= (*E*(*t*), *I*(*t*))^T^* and

$$B:=\left(\begin{array}{cc} \beta_{1} & \beta_{2}\\ 0 & 0 \end{array}\right),\;C:=\left(\begin{array}{cc} -\varepsilon & 0\\ \varepsilon & -\gamma \end{array}\right)$$ (3)

Therefore the next generation matrix with large domain *K*^†^ is calculated as

$$K=B{\left(-C\right)}^{-1}=\left(\begin{array}{cc} \frac{\beta_{1}}{\varepsilon }+\frac{\beta_{2}}{\gamma } & \frac{\beta_{2}}{\gamma }\\ 0 & 0 \end{array}\right)$$ (4)

Then the basic reproduction number is given by

$$R_{0}=\frac{\beta_{1}}{\varepsilon }+\frac{\beta_{2}}{\gamma }$$ (5)

Let

$$R_{01}:=\frac{\beta_{1}}{\varepsilon }\text{ and }R_{02}:=\frac{\beta_{2}}{\gamma }$$ (6)

be the reproduction numbers for the asymptomatic and symptomatic infection, respectively. Note that *R*_0_ = *R*_01_ + *R*_02_.

### 2.2 State-reproduction number for symptomatic cases

Here it should be noted that the disease can not be eradicated by quarantine of symptomatic cases if *R*_01_ > 1. In case that *R*_01_ < 1, we can define the *state-reproduction number* for symptomatic infectives, denoted by *T*, ([3], [13]) as

$$T:=\frac{R_{02}}{1-R_{01}}$$ (7)

Then the subcritical condition *R*_0_ < 1 is satisfied if and only if *T* < 1. The state-reproduction number can be interpreted as the average number of secondary cases of symptomatic infecteds produced by a primary symptomatic case during its entire course of infection without intermediate symptomatic case.

Suppose that *R*_01_ < 1 and we can reduce the reproductivity of symptomatic individuals by quarantine and social distancing. Let $\hat{q}$ be the reduction ratio of reproductivity of symptomatic cases. Then the number of secondary cases produced by a symptomatic infected individual becomes $\left(1-\hat{q}\right)R_{02}$, so the state-reproduction number becomes $\left(1-\hat{q}\right)T$. Then the critical reduction ratio for symptomatic cases is calculated as

$$q^{*}=1-\frac{1}{T}$$ (8)

and $\hat{q}>q^{*}$ is sufficient to guarantee the subcritical condition

$$R_{01}+\left(1-\hat{q}\right)R_{02}<1$$ (9)

where the left-hand side is called the *control reproduction number* under the prevention policy^‡^.

Even when *R*_01_ > 1, the above control strategy can work if *R*_01_ becomes less than unity by using general social distancing policy. If the social distancing policy reduces the basic reproduction number *R*_0_ to (1 −*r*) *R*_0_ and the reproductivity of asymptomatic case become subcritical; (1 − *r*) *R*_01_ < 1, the control state-reproduction number for symptomatic infectives associated with the reduction proportion *r* ∈ (0,1) is

$$T_{r}:=\frac{\left(1-r\right)R_{02}}{1-\left(1-r\right)R_{01}}$$ (10)

Then the critical reduction ratio for reproductivity of symptomatic cases under the social distancing policy is given by

$$q_{r}:=1-\frac{1}{T_{r}}$$ (11)

### 2.3 Testing and quarantine model

**Figure 5.**
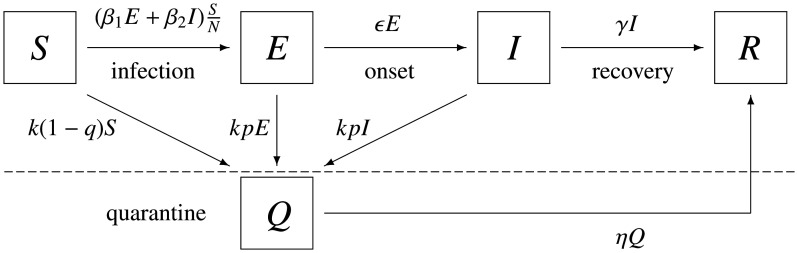
Transfer diagram for model (12).

Next here we consider a situation that susceptibles and infecteds are all assumed to be exposed to massive testing (PCR test) with testing rate *k* followed by case isolation. Let *p* ∈ (0,1) be the sensitivity of the test, and *q* ∈ (0,1) be the specificity of the test. Suppose that if the test reaction is positive, individuales are quarantined, no matter whether the reaction is pseudo or not. Let *Q* be the quaratined population. We assume that the quarantined individuals are excluded from the contact process. Then under the massive testing and quarantine strategy, the total dynamics is described as follows (see also Figure 5):

$$\begin{array}{l} \frac{dS}{dt}=-\left(\beta_{1}E+\beta_{2}I\right)\frac{S}{N}-k\left(1-q\right)S\\ \frac{dE}{dt}=\left(\beta_{1}E+\beta_{2}I\right)\frac{S}{N}-\left(\varepsilon +kp\right)E\\ \frac{dI}{dt}=\varepsilon E-\left(\gamma +kp\right)I\\ \frac{dR}{dt}=\gamma I+\eta Q\\ \frac{dQ}{dt}=k\left(1-q\right)S+kp\left(E+I\right)-\eta Q \end{array}$$ (12)

where *η* denotes the recovery rate for the quarantined population. Now we can assume that *N* + *Q* = 1. Using the next generation matrix method again, it is easy to see that the control reproduction number under the massive testing and quarantine policy is given by

$$R_{c}=\frac{\beta_{1}}{\varepsilon +kp}+\frac{\beta_{2}\varepsilon }{\left(\varepsilon +kp\right)\left(\gamma +kp\right)}$$ (13)

Let

$$R_{c1}:=\frac{\beta_{1}}{\varepsilon +kp}\text{ and }R_{c2}:=\frac{\beta_{2}\varepsilon }{\left(\varepsilon +kp\right)\left(\gamma +kp\right)}$$ (14)

be the control reproduction numbers for the asymptomatic and symptomatic infection, respectively. Note that *R_c_* = *R_c_*_1_ + *R_c_*_2_.

Note that *S* is monotone decreasing and *E*, *I* → 0 as *t* → +∞8 in both of models (1) and (12). That is, similar to the classical Kermack-McKendrick model without demography [3], there is no endemic steady state and the solution always converges to the disease-free steady state.

## 3 Results

### 3.1 Estimation of the baseline parameters

Let the unit time be 1 day. We set the average incubation period 1/*ε* to be 5 days [14]–[16], the average infectious period 1/*γ* to be 10 days [14], [17] and the average quarantine period 1/*η* to be 14 days [18]. That is, *ε* = 1/5 = 0.2, *γ* = 1/10 = 0.1 and *η* = 1/14 ≈ 0.07. We set the sensitivity *p* and the specificity *q* for testing to be 0.7 [19], [20] and 0.99 [20], respectively. Based on [2], we assume that the basic reproduction number in Japan is *R*_0_ = 2.6 (95%CI, 2.4–2.8), where CI denotes the credible interval.

In [10], it was estimated that 44% of secondary cases were infected during the presymptomatic state. Based on this estimation, we assume that *R*_01_ = 0.44*R*_0_ and *R*_02_ = 0.56*R*_0_. From (6), we have

$$\begin{array}{l} \beta_{1}=0.23\left(95\%\text{CI},0.21-0.25\right)\approx \varepsilon R_{01},\\ \beta_{2}=0.15\left(95\%\text{CI},0.13-0.16\right)\approx \gamma R_{02}. \end{array}$$ (15)

Note that the reason why *β*_1_ > *β*_2_ in spite of *R*_01_ < *R*_02_ is that *ε* = 0.2 is twice larger than *γ* = 0.1. Consequently, we obtain the baseline parameters as shown in Table 1.

**Table 1.** Description of each symbol in our simulation.

Symbol	Description	Value	Reference
*β*_1_	Asymptomatic infection rate	0.23 (95%CI, 0.21–0.25)	[15]
*β*_2_	Symptomatic infection rate	0.15 (95%CI, 0.13–0.16)	[15]
*p*	Sensitivity	0.7	[19], [20]
*q*	Specificity	0.99	[20]
1/*ε*	Average incubation period	5	[14]–[16]
1/*γ*	Average infectious period	10	[14], [17]
1/*η*	Average quarantine period	14	[18]
*R*_0_	Basic reproduction number	2.6 (95%CI, 2.4–2.8)	[2]
*R*_01_	Reproduction number for asymptomatic infection	0.44 *R*_0_	[10]
*R*_02_	Reproduction number for symptomatic infection	0.56 *R*_0_	[10]
*r*, *u*	Reduction proportion	0–1	-
*T_r_*	Control state-reproduction number	(see Figure 6)	[10]
*q_r_*	Critical reduction ratio	(see Figure 6)	[11]
*R_c_*	Control reproduction number	(see Figure 7)	[13]
*h*(*t*)	Positive predictive value	(see Figure 11)	[17]

### 3.2 Quarantine of symptomatic individuals under social distancing

**Figure 6.**
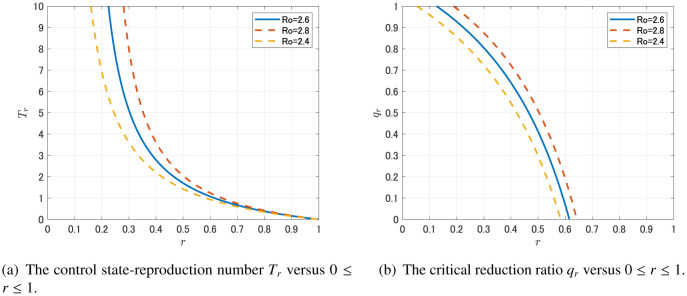
The dependence on *r* of the control state-reproduction number *T_r_* and the critical reduction ratio *q_r_* (which are given by (10) and (11), respectively).

We first consider the basic asymptomatic transmission model (1). From Table 1, we see that *R*_01_ = 0.44 *R*_0_ ≈ 1.14 (95%CI, 1.06–1.23) > 1. Hence, as stated in Section 2.2, we can not eradicate the disease only by quarantining symptomatic individuals. The control state-reproduction number *T_r_* and the critical reduction ratio *q_r_* under the social distancing policy that reduces *R*_0_ to (1 – *r*)*R*_0_, for 0 ≤ *r* ≤ 1 are plotted in Figure 6.

Figure 6 suggests us that if the social distancing leads to about 60% reduction of the contact rates (*r* = 0.6), then the disease can be eradicated without extra quarantine measure for symptomatic individuals. Moreover, it also suggests that even if the social distancing leads to relatively mild reduction of the contact rates, the disease can be eradicated with sufficient quarantine of symptomatic individuals. For instance, if *r* = 0.3 (30% reduction of the contact rates by social distancing), then *q_r_* = 0.80 (95%CI, 0.73–0.87), which implies that 80% reduction of the symptomatic individuals' contact rate by massive testing and quarantine could result in the eradication of the disease. Note that detection and quarantine of symptomatic individuals would be much easier than that of asymptomatic individuals. Thus, *q_r_* = 0.80 might not be an unrealistic goal.

### 3.3 Sensitivity of the control reproduction number

**Figure 7.**
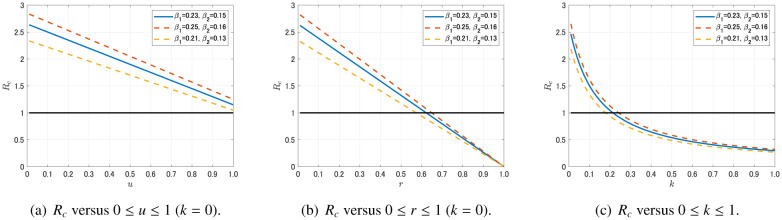
Sensitivity of the control reproduction number *R_c_* for parameters *u*, *r* and *k*.

We next change our focus from the asymptomatic transmission model (1) to the testing and quarantine model (12). We investigate the effect of each intervention strategy by observing the sensitivity of *R_c_*, which is defined by (13). First, we consider a quarantine of symptomatic individuals that results in reducing the symptomatic infection rate *β*_2_ to (1 − *u*)*β*_2_, where 0 ≤ *u* ≤ 1. In this case, the control reproduction number *R_c_* decreases linearly with increasing *u*, however, *R_c_* < 1 is not attained even if all symptomatic individuals are successfully quarantined (see Figure 7 (a)).

Next, we consider a social-distancing that results in reducing the infection rates *β_i_* to (1 − *r*)*β_i_*, where *i* = 1, 2 and 0 ≤ *r* ≤ 1. In this case, the control reproduction number *R_c_* decreases linearly with increasing *r*, and *R_c_* < 1 is attained for about 60% (*r* = 0.6) reduction of the infection rates (see Figure 7 (b)). This result is consistent with the result in Section 3.2, Figure 6 (a).

Finally, we consider a massive testing with quarantine that results in increasing the testing rate *k*. In this case, *R_c_* is monotone decreasing and convex downward for 0 ≤ *k* ≤ 1, and *R_c_* < 1 is attained for about *k* = 0.2 (see Figure 7 (c)). Note that *R_c_* is highly sensitive for small *k* since it is convex downward. This implies that the increasement of the testing rate would be an effective strategy to control the disease especially in countries with an originally low level of testing rate such as Japan.

### 3.4 Epidemic curves under the intervention

**Figure 8.**
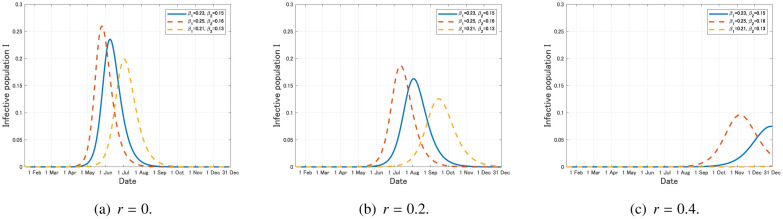
Time variation of the infective population *I* with the baseline parameters in Table 1 and the social distancing that reduces *β_i_* to (1 – *r*)*β_i_*, *i* = 1,2, where 0 ≤ *r* ≤ 1 (*k* = 0).

We next observe the epidemic curves of model (12) under each intervention. We assume that one infective individual is confirmed in Japan at *t* = 0 (15 January, 2020) and, for simplicity, there was no exposed, removed and quarantined individuals at *t* = 0. That is,

$$S\left(0\right)=1-\frac{1}{1.26\times 10^{8}},\;E\left(0\right)=0,\;I\left(0\right)=\frac{1}{1.26\times 10^{8}},\;R\left(0\right)=Q\left(0\right)=0$$ (16)

where 1.26×10^8^ implies the total population in Japan [21]. The epidemic curves of model (12) under the continued social distancing are displayed in Figure 8.

From Figure 8, we see that 40% reduction of the infection rates (*r* = 0.4) results in the drastic reduction of the epidemic size. However, we have to keep such a social distancing during the full period and it could largely affect the social and economic systems. Moreover, the recurrence of the epidemic could possibly occur if we stop the intervention on the way (see also Section 3.5).

**Figure 9.**
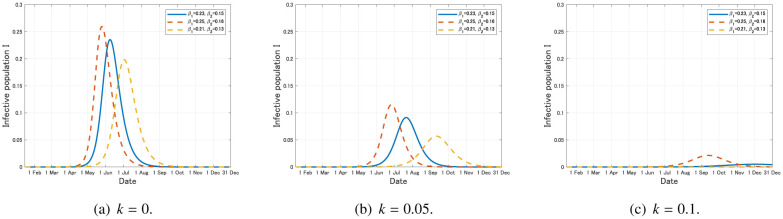
Time variation of the infective population *I* with the baseline parameters in Table 1 and the massive testing and quarantine.

The epidemic curves under the massive testing and quarantine are displayed in Figure 9.

We see from Figure 9 that increasing *k* up to 0.1 is sufficient for drastically reducing the infective population within this year. Since massive testing and quarantine would less affect the social and economic systems, to keep them for a long term could be one of the effective and realistic strategies.

### 3.5 Recurrence of the epidemic

We next consider the possibility of the recurrence of the epidemic in Japan after the social distancing, which started when the state of emerngency (SOE) was declared on 7 April, 2020. As in the previous subsection, we regard *t* = 0 as 15 January, 2020 and the initial condition is given by (16). We assume that the infection rates *β_i_* (*i* = 1,2) are reduced to (1 – *r*)*β_i_* (*i* = 1,2) with *r* = 0.8 (80% reduction of the contact rate, which was recommended by the Japanese government at April) during the period of social distancing, which starts from *t* = 83 (7 April, 2020) to some planned date t* > 83.

**Figure 10.**
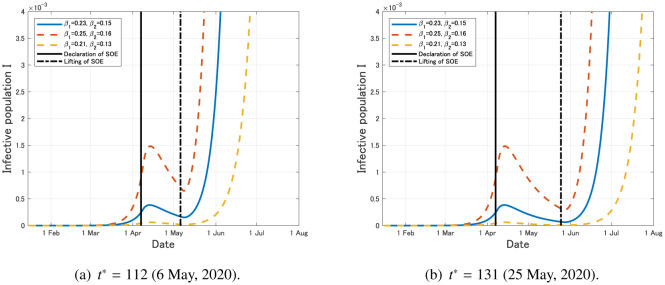
Time variation of the infective population *I* with the social distancing that leads to the 80% reduction of the contact rate during the period from *t* = 83 (7 April, 2020) to some planned date t* > 83 (*k* = 0).

First, we assume that the SOE is lifted on the originally planned date 6 May, 2020, that is, t* = 112. In this case, the exponential growth of the infective population *I* starts again after the lifting of SOE (see Figure 10 (a)).

Next, we assume that the SOE is lifted on the extended date 25 May, 2020, that is, t* = 131. Similar to the previous case, the exponential growth of the infected population *I* starts again after the lifting of SOE (see Figure 10 (b)).

From Figure 10, we can conjecture that the recurrence of the COVID-19 epidemic after the lifting of SOE is fully possible in Japan if the infection rates return to the original level after the lifting. To avoid this bad scenario, we should keep appropriate reduction of the contact rates even after the lifting of SOE and infected individuals must be tested and quarantined effectively, otherwise the second epidemic wave might cause a long-term damage to the social and economic systems.

## 4 Discussion

**Figure 11.**
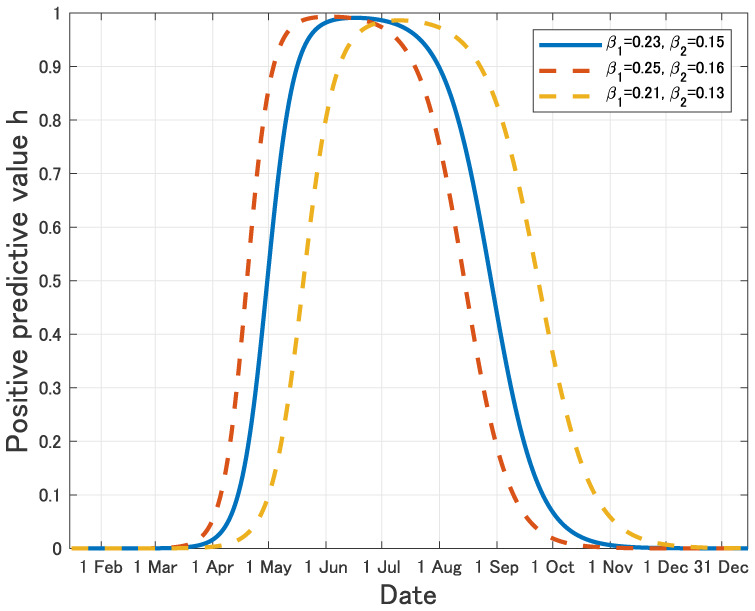
Time variation of the positive predictive value *h*(*t*) in our model.

As is usually pointed out as a weak point of testing, the positive predictive value (probability that tested positive individuals are really infected) for testing is very small as long as the prevalence is low, and so a lot of tested positive individuals are in fact not infected. If we calculate the positive predictive value by using our modelling, it is given as

$$h\left(t\right):=\frac{p\left(I+E\right)}{\left(1-q\right)S+pI+E}=\frac{\left(Tested\;positive\right)}{\left(Tested\;pseudo\text{-}positive\right)+\left(Tested\;positive\right)}$$ (17)

from which we know that it is very small in the early stage of epidemic (see Figure 11), hence many uninfected individuals will be quarantined because their test reaction is pseudo-positive, while many infecteds will be escaped from quarantine because of pseudo-negativeness.

This fact has been used to support the early control strategy such that instead of widespread testing, symptomatic individuals and asymptomatic individuals linked to infected local groups should be mainly targeted for testing, because their prior probability of positive is high and the positive predictive value is also high, so we can avoid quarantine of pseudo-positive individuals. However, as confirmed case numbers are rising, contact tracing is more difficult, many transmission routes that don't involve observed infection clusters appear, and so community spread in the big cities will be missed. If we have entered into such explosive phase of the epidemic, as is shown above, the massive testing could be a strong tool to prevent the disease as long as the positively reacted individuals will be effectively quarantined, no matter whether the positive reaction is pseudo or not.

In our simulation above, the control reproduction number is less than unity when the testing rate *k* is about 0.2 (20 percent per day), which seems to be too high in realistic situation if the host population is assumed to be national level. However, if once the number of daily produced symptomatic individuals is lowered by comprehensive social distancing policy, and the risk group can be visualized, the target community size is not so large, the masssive testing would be very effective. As is shown in Figure 9, the epidemic is largely mitigated even if *k* is 5 percent par day, because the control reproduction number is very sensitive with respect to small *k*. Since total population could be seen as a superposition of smaller communities, we could understand how testing and quarantine policy might be powerful to control the infectious disease.

Finally, we would like to point out a possible extension of our model. In the asymptomatic transmission model (1), we implicitly assume that all exposed individuals will finally become symptomatic, so they are becoming observable. On the other hand, it is reported that for covid-19 virus, there are many infecteds without symptom, who are unobservable infecteds as long as they are not tested. It is our future challenge to extend the basic model to examine the effect of existence of never symptomatic individuals.

## Footnotes

* T denotes the transpose of the vector

† Since the symptomatic state is not the birth-state of new infecteds, *K* is not the next generation matrix [3].

‡ Here we did not use more well-known term; *effective reproduction number* (ERN), because recently it has been used as a daily estimated (instantaneous) reproduction number based on the observed onset cases in news for COVID-19, although originally ERN simply implies a reproduction number under any immunization policy or interventions.
